# Lifestyle Factors and Energy Intakes with Risks of Breast Cancer among Pre- and Post- Menopausal Women in Taiwan

**DOI:** 10.3390/nu15183900

**Published:** 2023-09-07

**Authors:** Meng-Chuan Huang, Tz-Ting Huang, Hsin-Chun Feng, I-Chun Chen, Chiao-I Chang, Tsu-Nai Wang, Wen-Hung Kuo, Ming-Yang Wang, Li-Wei Tsai, Szu-Yi Li, Chiun-Sheng Huang, Yen-Shen Lu, Ching-Hung Lin

**Affiliations:** 1 Department of Nutrition and Dietetics, Kaohsiung Medical University Hospital, Kaohsiung Medical University, Kaohsiung 807, Taiwan; mechhu@kmu.edu.tw (M.-C.H.); melady1910128@gmail.com (T.-T.H.); 2 Department of Public Health and Environmental Medicine, College of Medicine, Kaohsiung Medical University, Kaohsiung 807, Taiwan; s370002000@yahoo.com.tw; 3 Department of Nutrition, National Taiwan University Hospital, Taipei 100, Taiwan; 107435@ntuh.gov.tw; 4 Graduate Institute of Oncology, National Taiwan University, Taipei 100, Taiwan; a00523@ntucc.gov.tw (I.-C.C.); s0929s@gmail.com (S.-Y.L.); 5 Department of Medical Oncology, National Taiwan University Hospital, Cancer Center Branch, Taipei 100, Taiwan; 6 Department of Oncology, National Taiwan University Hospital, Taipei 100, Taiwan; 7 Department of Public Health, College of Health Science, Kaohsiung Medical University, Kaohsiung 807, Taiwan; wangtn@kmu.edu.tw; 8 Department of Surgery, National Taiwan University Hospital, Taipei 100, Taiwan; wenhung003915@ntu.edu.tw (W.-H.K.); huangcs@ntu.edu.tw (C.-S.H.); 9 Department of Surgical Oncology, National Taiwan University Hospital, Cancer Center Branch, Taipei 100, Taiwan; mingyang@ntuh.gov.tw (M.-Y.W.); b91401028@ntu.edu.tw (L.-W.T.); 10 Department of Internal Medicine, National Taiwan University Hospital, Taipei 100, Taiwan

**Keywords:** Asian, breast cancer, basal metabolic rate, dietary pattern, pre-menopausal, post-menopausal

## Abstract

Although the incidence of invasive breast cancer (BC) among women in Asian is generally lower than that in Western countries, the incidence of BC has been on the rise in the past three decades in Asian countries. This hospital-based case-control study aimed to explore the relationship between dietary and metabolic factors and BC risk in pre- and post-menopausal women. We enrolled 285 patients with newly diagnosed BC at the National Taiwan University Hospital and 297 controls from the local community and hospital staff. Before receiving anticancer therapy, all patients with BC and control participants completed a 57-question semi-quantitative Food Frequency Questionnaire. For pre-menopausal women, plant-based factor scores rich in seeds and nuts, soy, fruits, and seaweeds correlated significantly with reduced BC risks, whereas menarche occurring at <12 years of age, reduced physical activity, and high-density lipoprotein <40 mg/dL were associated with increased BC risks. For post-menopausal women, plant-based dietary factor scores were also associated with reduced risks, whereas increased body mass index and energy intake levels correlated with increased BC risks. Diets rich in plant-based dietary patterns are protective against BC risk, regardless of menopausal status. Habitual physical activity is protective against BC risk among pre-menopausal Taiwanese women. Maintaining optimal weight and caloric intake is beneficial for reducing post-menopausal BC risk.

## 1. Introduction

Approximately 2.1 million female breast cancer (BC) cases were newly diagnosed in 2018 worldwide, and the incidence of cases and deaths in females were 24.2% and 15.0%, respectively [1]. Although the incidence of BC in most Asian countries (25.9–45.3%) is lower than that in Western countries (54.5–94.2%), it has increased rapidly in recent years [1]. The incidence of invasive BC in women in Asian countries is generally lower than that in Western countries [2]. However, its incidence has rapidly increased over the past three decades in Asian countries [3,4,5,6]. Invasive female BC in Taiwan is characterized by a striking increase in incidence and a relatively young median age (45–49 years) at diagnosis [3,7]. By contrast, the peak age of BC incidence in Western countries is 60–70 years, after menopause [8]. Our previous study showed the distinct pathologies of BC between East Asian and American women, as well as between East Asian Americans and White Americans, suggesting racial differences in biology [9].

The known risk factors for BC include reproductive status, genetics, lifestyle, ethnicity, and anthropometric characteristics [10]. Reports from the World Cancer Research Fund/American Institute for Cancer Research [11] and meta-analyses [10] also strongly suggested that obesity, represented by body mass index (BMI), increases the relative risk of post-menopausal BC. However, increase in BMI was inversely associated with the risk of pre-menopausal BC among Africans and Caucasians. By contrast, a significant positive association was observed among Asian women [12]. The unique positive association between BMI and BC risk in Asian women may suggest that dietary and metabolic factors play distinct roles in breast carcinogenesis.

Most case-control and prospective studies have found a positive association between high energy intake and BC risk, with the association being stronger in post-menopausal than in pre-menopausal women [13,14]. Among studies that investigated the metabolic effects of voluntary weight loss, a substantial reduction in the biomarkers of estrogen stimulation and inflammation was observed in both pre-menopausal and post-menopausal women following weight loss [15]. A positive energy balance is considered as excessive energy intake in relation to energy expenditure (resting energy expenditure or physical activity), rather than high energy intake per se. Previous studies have explored the three components of energy balance, including energy intake, energy expenditure, and body mass, in relation to BC risk, which were detected in pre- [16], post-menopausal women [17,18], or both [19,20], with the least favorable energy balance profile: high-energy intake, high BMI, and low physical activity.

In addition to energy intake or balance and dietary patterns, an alternative approach to evaluating dietary content is commonly used to assess the correlations between diet and cancer risk. A recent meta-analysis suggested a possible increased risk of BC associated with a Western dietary pattern, these associations were significant among post-menopausal but not pre-menopausal women; and a reduced risk with a prudent dietary pattern was significant among pre-menopausal but not post-menopausal women [21]. To the best of our knowledge, studies exploring multiple metabolic factors related to energy balance, dietary patterns, and BC risk in Asian populations are lacking. In this study, we aimed to investigate the interrelations among dietary patterns and metabolic profiles, including BMI, energy intake, and resting energy expenditure, and BC risks stratified by menopause status, using a hospital-based case-control study design.

## 2. Materials and Methods

### 2.1. Study Design and Participant Recruitment

This case-control study involving face-to-face interviews was conducted at the National Taiwan University Hospital (NTUH) from January 2011 to October 2017. Patients with malignant BC (*n* = 285) were included, in which BC diagnosis was confirmed with positive histological samples. Patients with malignant BC, in which BC diagnosis was confirmed with positive histological samples, were invited by physicians at outpatient clinics of the NTUH. The proportion of the eligible participants who agreed to enter the study (the response rate) was approximately 55%, with a total of 285 BC patients enrolled. Patients with other malignancies were excluded. Hospital- and visitor-based control participants were patients with benign breast tumors (*n* = 99) or healthy control participants (*n* = 198), who were recruited through flyers posted at the NTUH or in a nearby community. The response rate of the eligible control participants was approximately 85%, with a total of 99 participants with benign breast diseases and 197 healthy control participants enrolled.

The inclusion criterion for all study participants were female sex with ages ranging from 20 to 85 years. The health histories of all participants were obtained through questionnaire interviews, and participants with a history of other cancers or catastrophic illness including severe heart failure, renal failure, liver cirrhosis, or autoimmune diseases were excluded. Patients with complete information on body composition, clinical parameters, and dietary information, which was obtained through a 57-question semi-quantitative food-frequency questionnaire (FFQ), were included in the analysis. All participants recruited in this study signed a consent form, and the research protocol was approved by the ethics committee of National Taiwan University Hospital (201307001RINA).

### 2.2. Clinical Characters and Dietary Data Collection

During enrollment, participants completed an assisted questionnaire, including questions related to basal metabolic rate (BMR), which was evaluated using the Harris–Benedict equation, habitual smoking (≥1 time/week and for ≥6 months), drinking (≥1 time/week and for ≥6 months), and physical activity (≥30 min/week and for ≥6 months). The questionnaire also included demographic characters and known BC risk factors, such as age, body mass index (BMI) [22], menopausal status, educational level, and family history of breast and/or ovarian cancer.

A semiquantitative FFQ (44 items) initially validated for evaluating the diet of patients with type 2 diabetes [23], was further modified into a 57-item FFQ for surveying cancer patients. The reasons for such a modification were primarily to expand food items or food categories related to BC risks, which have been often addressed in epidemiological studies [24]. Before starting cancer therapy, all patients were interviewed by trained research assistants, to obtain dietary data using the FFQ, as well as cancer-related eating habits. Participants were asked questions regarding the frequency of their consumption of food items belonging to certain food groups over the previous six months, with nine frequency options ranging from “almost never” to “four to six times per day”. After completing the dietary survey, participants were referred to a registered dietician specializing in cancer nutrition to confirm the dietary information obtained from the FFQ and also for further counseling.

Overall, 23 of the 57 questions were semiquantitative, and food models resembling standard portion sizes for food groups were used to enhance the accuracy of the diet-related information. All information collected was used to estimate the daily intake of each food group. The frequencies were converted into the number of servings per week and multiplied by the declared portion size. During our study period, the daily nutrient intake of each participant was estimated based on the food database established by the Food and Drug Administration in Taiwan https://consumer.fda.gov.tw/Food (accessed on 20 October 2019). Energy, macronutrients, and fiber were calculated in kcal or g per day.

### 2.3. Statistical Analysis

**Figure 1:**
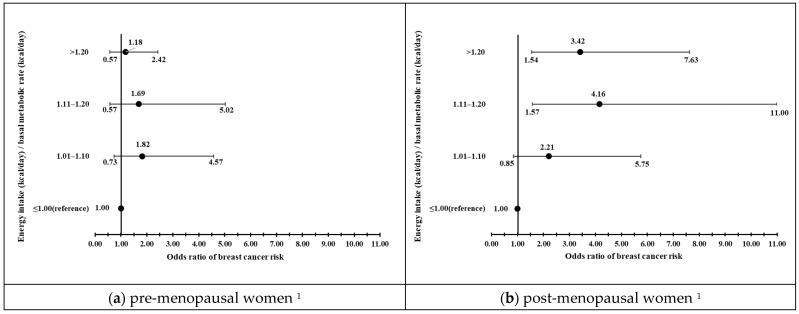

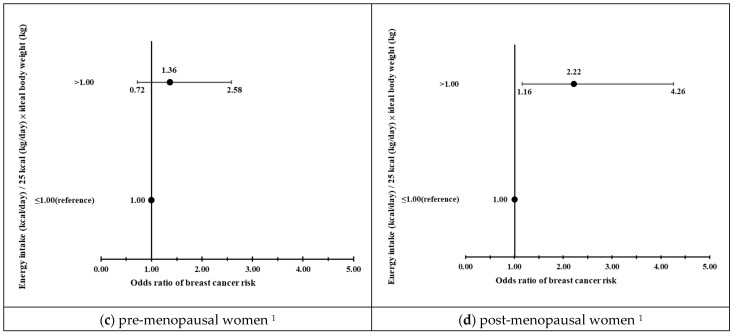
Energy intakes relative to basal metabolic rate (BMR) or 25 kcal/kg/day as recommended for energy intakes for patients with cancer [26] in pre- and post-menopausal women. (**a**,**b**) Roles of energy intakes relative to BMR in risk of breast cancer (odds ratio (OR)) in pre- and post-menopausal women. (**c**,**d**) Roles of energy intake relative to 25 kcal/kg/day in risk of breast cancer (OR) in pre- and post-menopausal women. ^1^ Multivariable logistic regression analysis was used to examine the associations between the ratio of total energy intake (TEI)/BMR or TEI/recommended energy intake and BC risks. Confounders included age, BMI, education year, family history of breast cancer, smoking, alcohol drinking, physical activity, age at menarche, triglyceride, HDL-C, and energy intake among pre- and post-menopausal women.

Descriptive data were expressed as mean ± standard deviation for continuous or *n* (%) for categorical variables. Chi-square tests or t-tests were used to assess differences in demographic and clinical characteristics between the case and control groups. A factor analysis, a multivariate statistical technique used to reduce the complexity of diet into fewer independent factors, was used to identify dietary patterns among the study participants from the FFQ responses. The factors were rotated using orthogonal transformation (varimax rotation), to maintain uncorrelated factor variables called principal components or patterns. The factor scores for each dietary pattern and individual were calculated by summing the intakes of food items or groups weighted by their factor loadings. Dietary patterns were named according to food items or groups that presented high factor loadings. In this study, cut-off points of equal or greater than 0.3 were used as an indication to show that food items or groups contributed prominently to a specific dietary pattern. Three dietary patterns were identified: high fat and sugar, plant-based food, and animal protein. Multiple logistic regression analyses were performed to examine the independent association between nutrient intakes or dietary patterns and risks of BC, by adjusting for the following confounding factors: age, BMI, total caloric intake (<1000, 1000–1199, 1200–1399, ≥1400 kcal/day), alcohol drinking (yes, no), smoking (yes, no), triglyceride (<150, >150 mg/dL), high-density lipoprotein cholesterol (HDL-C) (<40, ≥40 mg/dL), physical activity (yes, no), education year (<12, ≥12 years), age at menarche (<12, ≥12 year of age), and family history of BC (yes, no). For TG and HDL, determination of cut-offs was based on Third Report, National Cholesterol Education Program (ATP III) Executive Summary [25]. HDL <40 mg/dL was defined as low HDL and triglycerides <150 mg/dL as elevated triglycerides. Levels of BMR were calculated using the Harris–Benedict equation, and recommended energy intake level (25–30 kcal/kg/day) was based on ESPEN guidelines on nutrition in cancer patients [26]. Intakes of energy divided by BMR or 25 kcal/kg/day were further applied to explore to what extent energy intakes exceeding BMR or recommended energy levels correlated with risks of BC (Figure 1). All statistical analysis was performed using IBM SPSS Statistics for Windows, version 22 (IBM Corp., Armonk, NY, USA), and the statistical significance was set at *p* < 0.05.

## 3. Results

The demographic and clinical characteristics of the women with BC and controls are presented in Table 1 (*n* = 582). Patients with BC were more likely to be older and have fewer years of education (≤12 years). A greater proportion of patients with BC were married (72.3% vs. 61.6%) and in the post-menopausal stage (53.0% vs. 38.0%) compared with the control group. Regarding lifestyle-related characteristics, patients with BC were more likely to be physically inactive and smokers (both *p* < 0.05). For anthropometric measures, patients with BC showed worse anthropometric measures, including higher body weight (58.9 ± 10.1 kg vs. 55.8 ± 9.1 kg, *p* < 0.001) and higher BMI categories (<23.0 (50.5% vs. 63.5%), 23.0–27.49 (31.9% vs. 28.4%), ≥27.5 kg/m^2^ (17.5% vs. 8.1%), *p* = 0.001), between the two groups. Interaction effects were observed between menopausal state and BMI <27.5 vs. ≥27.5 kg/m^2^ (*p* < 0.01). With regard to blood lipids, patients with BC showed significantly higher levels of triglycerides (115.6 ± 78.9 mg/dL vs. 96.1 ± 49.9 mg/dL, *p* < 0.001) and lower HDL (54.5 ± 12.0 vs. 58.4 ± 12.5 mg/dL, *p* < 0.001).

Three major patterns were identified in the factor analysis: high-fat, high-sugar, plant-based, and high-animal protein dietary patterns (Supplementary Table S1). Principal component analysis was performed on the 57 food groups using varimax rotation. The Kaiser–Meyer–Olkin sampling adequacy test score was 0.826, which was considered meritorious for this FFQ. The rotated factor loadings for food groups with an absolute value of ±0.3 for three factors were high-fat and sugar, plant-based food, and animal protein. These three factors are characterized as follows: The high-fat, high-sugar pattern was characterized by a high intake of fast foods, fried foods, beverages, starchy foods, processed foods, staple foods, internal organs, canned meats, sugar substitutes, and frequent eating out habits. The plant-based pattern was characterized by the high intake of soy products, mushrooms, seaweed, root vegetables, fruits, seeds, nuts, fermented products, processed wheat/gluten products, low-calorie deserts, and yogurt. High animal protein patterns were characterized by the high intake of animal proteins, including fish, dried fish products, red meat, white meat, seafood, fatty meat, skin, and eggs.

The identified risk factors for BC in pre- and post-menopausal women are shown in Table 2. Upon adjusting for confounding factors in model 1, plant-based dietary patterns significantly correlated with reduced risk of BC (Tertile 2: OR = 0.40, 95% CI = 0.20–0.77; tertile 3: OR = 0.23, 95% CI = 0.11–0.49), whereas no exercise habits (odds ratio (OR) = 2.10, 95% confidence interval (CI) = 1.21–3.65), and menarche occurring at ≤12 years of age (OR = 2.49, 95% CI = 1.10–5.64) and HDL <40 mg/dL (OR = 6.74, 95% CI = 1.76–25.83) were related to augmented BC risk among pre-menopausal women. Similarly, among post-menopausal women, second and third tertile levels of plant-based dietary pattern score (Tertile 2: OR = 0.43, 95% CI = 0.20–0.93; tertile 3: OR = 0.28, 95% CI = 0.12–0.65) were associated with decreased BC risk in a dose-dependent manner (*p* for trends = 0.002). Other significant and positive risk factors included higher BMI (OR = 1.10, 95% CI = 1.01–1.20) and greater energy intake levels (1000–1199, 1200–1399, ≥1400 kcal/day vs. <1000 kcal/day, *p* < 0.001).

The roles of BMR in BC risk were further explored, and the results showed that total energy intake (TEI)/BMR ratio reaching 1.11–1.20- and >1.20-fold of BMR, respectively, were associated with 4.16- (95% CI = 1.57–11.00) and 3.42-fold (95% CI = 1.54–7.63) increases in BC risks among post-menopausal women. Furthermore, checking the balance between TEI and recommended energy intake for patients with cancer (25 kcal/kg/day × ideal body weight) [26] showed that positive levels were associated with augmented BC risks (OR = 2.22, 95% CI = 1.16–4.26) among post-menopausal women. By contrast, neither TEI/BMR nor the balance between TEI/recommended energy intake for patients with cancer were related to BC risk among pre-menopausal women (Figure 1).

## 4. Discussion

In this study, pre-menopausal women who reported no habitual physical activity and HDL <40 mg/dL were significantly correlated with increased BC risk. Among post-menopausal women, those who had a caloric intake of 1200–1399 kcal/day, ≥1400 kcal/day in reference to those eating <1200 kcal/day, had significant and dose-responsive positive risks for BC; moreover, those with increased BMI also correlated positively with BC risk [27]. This study highlights the differences in dietary- and metabolism-related risk factors between pre- and post-menopausal East Asian women.

The relationship between physical activity and BC risk has been evaluated in >30 epidemiological studies [28,29]. A previous study revealed that high levels of physical activity may reduce BC incidence by 30–70% [30]. One possible biological mediator is HDL-C. Physical activity is known to improve cardiovascular system function, such as lowering blood pressure and triglyceride, augmenting VO_2_ max, and elevating HDL-C levels in patients with BC [31]. A recent meta-analysis, including prospective studies and elimination of preclinical bias, verified a significant inverse correlation between cholesterol, more specifically HDL-C and BC risk [32]. The role of HDL-C has been supported by experimental studies demonstrating its antioxidant and anti-inflammatory properties [33]. Furthermore, Emaus et al. [34] found that 204 healthy pre-menopausal women with greater leisure-time physical activity had lower estradiol level. This relationship remained unchanged when the obesity component was excluded, suggesting that the protective effect of physical activity was not completely mediated by changes in adiposity. Similar to our findings in pre-menopausal women, the Atherosclerosis Risk in Communities Study recruited 7575 female members during a follow-up period of 1987 to 2000, and 359 cases of incident BC were ascertained. Upon adjusting for possible confounding factors, an association of HDL-C <50 mg/dL with incident BC was not observed in the total participants, whereas an association of HDL-C <50 mg/dL with incident BC risk (HR = 1.67 (95% CI = 1.06–2.63) was observed in women who were pre-menopausal at baseline. This may be partially explained within the context of the hormonal regulation of BC. It is well established that high endogenous estrogen concentrations serve as a robust risk factor for BC in pre-menopausal women [35]. Levels of dehydroepiandrosterone, a precursor of estrogen in women, observed during the premenopausal period were inversely correlated with HDL-C [36]. As menopause leads to a reduction in endogenous estrogen concentration, the probable effect of HDL-C in decreasing the activity of endogenous estrogen would thus be attenuated.

Recent meta-analyses have extensively examined the relationship between BMI and BC [37]. Being overweight and obese appear to be extremely consistent with increased BC risk in post-menopausal women; however, controversy exists regarding their impact on pre-menopausal women [12,37]. In our investigation, through the stratification of menopause status, we found that the distribution of different BMI categories (<23.0 (65.7% vs. 67.9%), 23.0–27.49 (23.9% vs. 25.5%), ≥27.5 (10.4% vs. 6.5% between pre-menopausal cases and controls was not different (*p* = 0.448). For post-menopausal participants, one unit increase in BMI correlated with a 10% increase in risk (OR = 1.10, 95% CI = 1.01–1.20, *p* = 0.023).

There is strong interest in exploring ways to disrupt the obesity–BC link, and one key approach may be through diet modification. Animals on a calorie-restricted diet were shown to have a reduced occurrence of spontaneous mammary tumors [38], tumor multiplicity, and tumor burden [39]. An earlier study involving female patients hospitalized for anorexia nervosa in their earlier life reported a 50% statistically significant reduction in BC risk [40]. Epidemiological studies in humans generally do not support an association between dietary energy intake alone and the risk of BC, although some studies have suggested a more complex interplay between measures of energy intake, physical activity, and body size. A positive energy balance, whether generated by increased energy intake or decreased energy expenditure, or indicated by excess body weight, adversely influences the risk of BC. Among the post-menopausal participants in this study, both BMI and TEI levels were significantly correlated with increased BC risk (*p* trend < 0.001). As we further explored the extent that energy levels augmented BC risk, increased ratios (>1.20, 1.11–1.20, 1.10–1.00 vs. 1.00; *p* for trend = 0.002) of actual energy intake divided by BMR estimated using the Harris–Benedict equation or TEI exceeding 25/kg ideal body weight/day (OR = 2.22, 95% CI = 1.16–4.26) correlated significantly with BC risk in post- menopausal patients, but not in pre- menopausal women. In a U.S. cohort (*n* = 590 women) followed up for 15 years at an earlier time, BC risk was reported to be more than double for every 500 kcal/d increment in TEI [41]. Our findings are consistent with recent studies reporting positive relationships between energy density or intake and BC risk in the United States [42] and Spain [43]. Thus, energy imbalance may affect BC risk by affecting the circulating levels of insulin, insulin growth factor, and sex hormone-binding globulin [44,45]. A metabolic profile reflecting a combination of risk factors associated with an increased risk of cancer (obesity, Western diet, low physical activity) may provide a growth-promoting environment for cells, particularly neoplastic cells, possibly through influences on insulin resistance [46].

A meta-analysis of 75 studies investigating food groups and the risk of BC showed an inverse association between vegetables, cheese, fruit, and soy. The subgroup analyses also did not detect a significant interaction with menopausal status. By contrast, the positive association between red and processed meats and subgroup analyses showed a stronger association with BC risk in European countries and post-menopausal women [24]. A more recent meta-analysis including 15 cohort and 34 case-control studies revealed that the consumption of fruits and vegetables was associated with a 29% lower risk of BC (RR = 0.71 (0.55, 0.93); RR = 0.71 (0.53, 0.95)); however, no significant association was found between meat, soy foods, and green tea consumption and BC risk. As for the dietary pattern, high adherence to a healthy dietary pattern was associated with a 38% reduction in BC risk (RR = 0.62 (0.44, 0.88; RR = 0.49 (0.27, 0.87)), while high adherence to an unhealthy dietary pattern was associated with a 44% increased risk (RR = 1.44 (1.06, 1.96)). Subgroup analyses according to menopausal status or geographic region have not yet been reported [47]. In our study, plant-based food patterns (vegetables, fruits, seaweeds, mushrooms, soy, etc.) remained correlated with reduced BC risk in both pre- and post-menopausal women after adjusting for ample confounding factors, revealing the important roles of healthy diets in reducing BC risk in East Asia.

Our data may be limited by the following assessment aspects. First, our study sample size was small and may not be applicable to other Asian populations. Larger ethnically matched studies might help to clarify whether this association can be found in other Asian and Chinese populations. Second, this study had a case-control design; thus, we could not confidently determine the cause-and-effect relationship between lifestyle factors and BC risk. The most commonly cited disadvantage in case-control studies is the potential for selection bias, recall bias, and temporality. In our study, our control participants included those with benign breast tumors and healthy volunteers recruited near the hospital or from a nearby community. Our controls may not represent a typical healthy control population. People volunteering their data for this study (*n* = 198, healthy subjects) or those seeking medical attention (*n* = 99, subjects with benign tumors) for cancers may have a particularly high level of health motivation, which may potentially have affected accurate estimate of the exposure distribution of the source population, and selection bias would result. In order to clarify our results, we further performed a sensitivity analysis (case = 285, healthy control = 198) by excluding those with benign tumors (Supplementary Table S2), and the results remained unchanged. Administration of FFQs often has an inherent recall bias, because individuals are asked to report what they remember about their previous intake, and the intake of food was generally long before the administration of the FFQ. Additionally, the accuracy of FFQs can also be influenced by intentional misreporting of their consumption of certain foods or affected by known disease status. In this study, assessments of life-style exposures (diet, physical activities, smoking … etc.) were a single assessment prior to diagnosis with BC and may not be representative of lifetime exposures related to disease progression. Although we tried to minimize the effect of the disease by performing the questionnaire survey before the BC diagnosis, our one-time exposure assessment may still have partially contributed to misclassification or some bias of our results. Third, the estimation of TEI by applying an FFQ is difficult and imperfect, and the possibility of errors with respect to the measurement of diet and calculation of energy intake may cause some variations. Furthermore, underreporting of energy intake has typically been detected in obese people [48], and the residual confounding effects of this error tend to attenuate the estimated risks. Fourth, our question regarding habitual physical activity was somewhat limited, such that the questionnaire asked only about the average time spent in a week over the past six months. Finally, due to the limited sample size, the heterogeneous effects on BC risk from hormone receptor status were not evaluated in this study.

## 5. Conclusions

In conclusion, the results of this study suggest that habitual physical activity and optimal HDL-C concentrations are protective against BC risk in pre-menopausal women in Taiwan, and maintenance of optimal weight and energy intake is beneficial for reducing the risk of BC in post-menopausal women. A plant-based dietary pattern may protect against BC risk in both pre- and post-menopausal women. The mechanisms that modulate these lifestyle factors against BC risk before or after menopause warrant further investigation.

## Figures and Tables

**Table 1:** Demographic and clinical characteristics of participants with breast cancer and controls (*n* = 582) ^1^.

Characteristics	Control (*n* = 297)	Case (*n* = 285)	*p* ^2^	OR (95% CI) ^3^
Demographic and clinical characters				
Age	50.5 ± 12.1	55.7 ± 10.2	<0.001	
Education (year)				
>12	222 (75.0)	158 (55.4)		1
≤12	74 (25.0)	127 (44.6)	<0.001	2.41 (1.70–3.43)
Marriage status				
Unmarried or other	114 (38.4)	79 (27.7)		1
married	183 (61.6)	206 (72.3)	0.006	1.62 (1.15–2.30)
Family history of breast cancer				
No	248 (83.5)	241 (84.6)		1
Yes	49 (16.5)	44 (15.4)	0.727	0.92 (0.59–1.44)
Smokers				
No	287 (96.6)	263 (92.3)		1
Yes	10 (3.4)	22 (7.7)	0.021	2.40 (1.12–5.16)
Alcohol drinking				
No	285 (96.0)	264 (92.6)		1
Yes	12 (4.0)	21 (7.4)	0.083	1.89 (0.91–3.92)
Physical activity				
Yes	160 (53.9)	116 (40.7)		1
No	137 (46.1)	169 (59.3)	0.001	1.70 (1.23–2.36)
Weight	55.8 ± 9.1	58.9 ± 10.1	<0.001	
BMI (kg/m^2^)				
<23.0	188 (63.5)	144 (50.5)		1
23.0–27.5	84 (28.4)	91 (31.9)		1.41 (0.98–2.04)
≥ 27.5	24 (8.1)	50 (17.5)	0.001	2.72 (1.60–4.63)
Age at menarche (year)				
>12	267 (90.2)	250 (87.7)		1
≤12	29 (9.8)	35 (12.3)	0.339	1.29 (0.77–2.17)
Oral contraceptive				
No	185 (94.9)	218 (93.6)		1
Yes	9 (4.6)	10 (4.3)	0.356	0.87(0.37–2.33)
Menopausal status				
Pre-menopausal	184 (62.0)	134 (47.0)		1
Post-menopausal	113 (38.0)	151 (53.0)	<0.001	1.84(1.32–2.55)
Menopausal status × BMI				
Pre-menopausal × BMI < 27.5	172 (58.1)	120 (42.1)		1
Pre-menopausal × BMI ≥ 27.5	12 (4.1)	14 (4.9)		1.67(0.75–3.74)
Post-menopausal × BMI < 27.5	100 (33.8)	115 (40.4)		1.65(1.16–2.35)
Post-menopausal × BMI ≥ 27.5	12 (4.1)	36 (12.6)	<0.001	4.30(2.15–8.60)
Receptor type				
ER+, HER2+		27 (11.3)		
ER+, HER2−		172 (72.0)		
ER−, HER2+		21 (8.8)		
ER−, HER2−		19 (7.9)		
Blood lipid				
Triglyceride (mg/dL)	96.1 ± 49.9	115.6 ± 78.9	<0.001	
<150	259 (87.5)	230 (80.7)		1
≥150	37 (12.5)	55 (19.3)	0.025	1.67(1.06–2.63)
Cholesterol (mg/dL)	188.5 ± 39.1	189.4 ± 37.8	0.776	
<200	190 (64.2)	192 (67.4)		1
≥200	106 (35.8)	93 (32.6)	0.420	0.87(0.62–1.22)
HDL-C (mg/dL)	58.4 ± 12.5	54.5 ± 12.0	<0.001	
≥40	283 (95.6)	260 (91.2)		1
<40	13 (4.4)	25 (8.8)	0.033	2.09(1.05–4.18)
LDL-C (mg/dL)	111.4 ± 33.1	113.2 ± 31.4	0.495	
<130	217 (73.3)	202 (70.9)		1
≥130	79 (26.7)	83 (29.1)	0.513	1.13(0.79–1.62)
Nutrient intakes ^4^				
Total Energy (kcal/day)	1365.2 ± 496.0	1362.3 ± 403.5	0.939	
Protein (g/day)	51.4 ± 23.9	47.9 ± 15.0	0.021	

Abbreviation: OR, odds ratio; 95% CI, 95% confidence interval; BMI, body mass index; HDL-C, high-density lipoprotein-cholesterol; LDL-C, low density lipoprotein-cholesterol; ER+, estrogen receptor positive; ER−, estrogen receptor negative; PR+, progesterone receptor positive; PR−, progesterone receptor negative. ^1^ Data are presented as mean standard deviation or *n* (%). ^2^ Chi-square or student *t*-test were used to investigate the differences between cases and control groups. ^3^ Crude OR was performed with a simple logistic regression analysis. ^4^ Macronutrient distribution is expressed as % of energy.

**Table 2:** Relations between clinical- and lifestyle-related factors and risks of breast cancer in pre- and post-menopausal women ^1^ (*n* = 582).

	Pre-Menopausal			Post-Menopausal		
	No. of Control/Case	Model 1 ^2^ aOR (95% CI)	*p*	No. of Control/Case	Model 2 ^3^ aOR (95% CI)	*p*
BMI (kg/m^2^)	184/134	0.98 (0.91–1.07)	0.679	113/151	1.10 (1.01–1.20)	0.023
Physical activity						
Yes	89/46	1		71/70	1	
No	95/88	2.10 (1.21–3.65)	0.008	42/81	1.58 (0.88–2.84)	0.122
High-fat & sugar dietary pattern						
Tertile 1	30/34	1		61/69	1	
Tertile 2	58/48	0.91 (0.44–1.89)	0.809	35/53	1.33 (0.70–2.53)	0.378
Tertile 3	96/52	0.70 (0.34–1.45)	0.336	17/29	1.25 (0.56–2.78)	0.589
*p* for trend ^4^		0.287			0.451	
Plant-based dietary pattern						
Tertile 1	58/58	1		24/54	1	
Tertile 2	63/40	0.40 (0.20–0.77)	0.007	39/52	0.43 (0.20–0.93)	0.033
Tertile 3	63/36	0.23 (0.11–0.49)	<0.001	50/45	0.28 (0.12–0.65)	0.003
*p* for trend ^4^		<0.001			0.002	
Animal protein dietary pattern						
Tertile 1	64/37	1		41/52	1	
Tertile 2	63/44	1.18 (0.60–2.30)	0.631	40/47	0.85 (0.43–1.66)	0.625
Tertile 3	57/53	1.44 (0.69–3.03)	0.331	32/52	0.99 (0.47–2.07)	0.980
*p* for trend ^4^		0.396			0.922	
Age at menarche (year)						
>12	160/112	1		107/138	1	
≤12	23/22	2.49 (1.10–5.64)	0.029	6/13	2.18 (0.70–6.75)	0.178
Triglyceride (mg/dL)						
<150	174/117	1		85/113	1	
≥150	9/17	2.26 (0.80–6.40)	0.126	28/38	0.55 (0.27–1.11)	0.092
HDL-C (mg/dL)						
≥40	179/120	1		104/140	1	
<40	4/14	6.74 (1.76–25.83)	0.005	9/11	0.85 (0.28–2.55)	0.766
Smoking						
No	178/121	1		109/142	1	
Yes	6/13	1.08(0.30–3.89)	0.910	4/9	1.22(0.29–5.16)	0.791
Alcohol drinking						
No	180/121	1		105/143	1	
Yes	4/13	4.51(1.18–17.29)	0.028	8/8	0.95(0.27–3.41)	0.943
Energy intake (kcal/day)						
<1000	34/22	1		27/22	1	
1000–1199	33/19	1.20 (0.47–3.06)	0.699	32/33	1.92 (0.80–4.58)	0.142
1200–1399	32/25	1.91 (0.76–4.79)	0.169	22/39	4.06 (1.60–10.30)	0.003
≥1400	85/67	1.68 (0.70–4.02)	0.241	32/57	5.55 (2.04–15.11)	0.001
*p* for trend ^4^		0.192			<0.001	

Abbreviations: aOR, adjusted odds ratio; BMI, body mass index; HDL-C, high-density lipoprotein-cholesterol. ^1^ Data are expressed as OR (95% confidence interval (CI)), and *p* < 0.05 was considered significantly different. ^2^ Multivariable logistic regressions were used to examine the associations between the tertile levels of three dietary patterns and breast cancer risk, upon adjusting for age, BMI, education year, family history of breast cancer, smoking, alcohol drinking, physical activity, age at menarche, triglyceride, HDL-C, and energy intake among pre-menopausal women. ^3^ Multivariable logistic regressions were used to examine the associations between the tertile levels of three dietary patterns and breast cancer risk, upon adjusting for age, BMI, education year, family history of breast cancer, smoking, alcohol drinking, physical activity, age at menarche, triglyceride, HDL-C, and energy intake among post-menopausal women. ^4^ *p* for trend was performed through simple linear regression analysis.

## Data Availability

The data presented in the study are available on request from the corresponding author.

## Supplementary Materials

The following supporting information can be downloaded at: https://www.mdpi.com/article/10.3390/nu15183900/s1, Supplementary Table S1: Factor loading matrix for major dietary patterns identified by factor analysis (*n* = 582); Supplementary Table S2: Association of clinical- and lifestyle-related factors in breast cancer patients (*n* = 285) and controls (*n* = 198) in Taiwan according to menopausal status.
