# Supplementary material for: Lifestyle Factors and Energy Intakes with Risks of Breast Cancer among Pre- and Post- Menopausal Women in Taiwan
Source: Nutrients. 2023 Sep 7;15(18):3900. doi: 10.3390/nu15183900 (PMC10534793; doi:10.3390/nu15183900)
Supplement: Supplementary file 1 [file nutrients-15-03900-s001.zip › nutrients-2551780-supplementary.pdf]

Supplementary Table S1. Factor loading matrix for major dietary patterns identified by factor analysis (n = 582)

Food items	Factor 1	Factor 2	Factor 3
	**High- fat and high sugar**	**Plant-based food**	**Animal protein**
Fast foods	0.657	-	-
Fried foods	0.650	-	-
Bubble tea	0.624	-	-
Sugar-containing beverages	0.559	-	-
Starchy/thickened soup and foods	0.545	-	-
Eating out	0.538	-	-
Fried dumplings	0.531	-	-
Processed sea food product	0.515	-	-
Can juice	0.499	-	-
Processed meats	0.484	-	-
Processed dairy products	0.479	-	-
Sauce use	0.476	-	-
Dark-coloured vegetables	- 0.465	-	-
Low nitrogen staple foods	0.465	-	-
internal organ	0.459	-	-
Steam dumplings	0.424	-	-
Light-coloured vegetables	- 0.345	-	-
Canned meats	0.340	-	-
Sugar substitute use	0.305	-	-
Tea	-	-	-
Bread	-	-	-
Whole Milk/cheese	-	-	-
Cakes and cookies	-	-	-
Fresh fruit juice	-	-	-
Mushrooms	-	0.589	-
Soybean	-	0.572	-
Seaweed	-	0.568	-
Root vegetables	-	0.555	-
Fresh fruit	-	0.513	-
Seeds and nuts	-	0.508	-
Soy products	-	0.498	-
Fermented products	-	0.456	-
Processed wheat/gluten products	-	0.383	-
Low calorie desserts and snacks	-	0.361	-
Yogurt	-	0.349	-
Pickled vegetables	-	0.347	-
Light milk/cheese	-	-	-
Marine fish	-	-	0.685
Freshwater fish	-	-	0.646
Red meats	-	-	0.638

White meats	-	-	0.623
Seafood	-	-	0.472
Dried fish products	-	-	0.462
Fatty meats and skin	-	-	0.417
Processed Soy products	-	-	- 0.396
Eggs	-	-	0.315
Chinese staples	-	-	-

Omitted from the table were food items or groups with factor loadings < ± 0.30 for all dietary pattern

Supplementary Table S2. Association of clinical-and lifestyle-related factors in breast cancer patients (n=285) and controls (*n* = 198) in Taiwan according to menopausal status. [1]

	Pre-menopausal			Post-menopausal		
	No. of Control/Case	Model 1 [2] aOR (95% CI)	p	No. of Control/Case	Model 2 [3] aOR (95% CI)	p
**BMI (kg/m²)**	117/134	0.93(0.85-1.03)	0.160	81/151	1.11(1.01-1.23)	0.030
**Physical activity**						
Yes	57/46	1		50/70	1	
No	60/88	2.23(1.12-4.43)	0.023	31/81	1.31(0.69-2.49)	0.416
**High- fat & sugar dietary pattern**						
Tertile 1	20/34	1		41/69	1	
Tertile 2	27/48	1.96(0.78-4.93)	0.152	27/53	1.19(0.58-2.44)	0.627
Tertile 3	70/52	1.11(0.46-2.67)	0.815	13/29	1.11(0.46-2.71)	0.812
P for trend[4]		0.954			0.741	
**Plant-based dietary pattern**						
Tertile 1	40/58	1		16/54	1	
Tertile 2	40/40	0.49(0.22-1.10)	0.085	27/52	0.45(0.19-1.05)	0.064
Tertile 3	37/36	0.24(0.10-0.62)	0.003	38/45	0.26(0.11-0.65)	0.004
P for trend[4]		0.003			0.004	
**Animal protein dietary pattern**						
Tertile 1	38/37	1		30/52	1	
Tertile 2	39/44	1.15(0.50-2.65)	0.738	26/47	0.86(0.40-1.84)	0.692
Tertile 3	40/53	1.33(0.55-3.24)	0.530	25/52	0.76(0.33-1.74)	0.518
P for trend[4]		0.530			0.515	
**Age at menarche (year)**						
> 12	97/112	1		75/138	1	
≤ 12	19/22	2.48(0.96-6.38)	0.060	6/13	1.71(0.54-5.42)	0.359
**Triglyceride (mg/dL)**						
< 150	111/117	1		59/113	1	
≥ 150	5/17	3.03(0.74-12.34)	0.122	22/38	0.50(0.23-1.07)	0.074
**HDL-C (mg/dL)**						
≥ 40	113/120	1		74/140	1	
< 40	3/14	6.16(1.21-31.30)	0.028	7/11	0.59(0.19-1.88)	0.375
**Smoking**						
No	116/121	1		121/142	1	
Yes	1/13	1.52(0.13-17.53)	0.736	13/9	3.43(0.36-33.05)	0.287
**Alcohol drinking**						
No	115/121	1		77/143	1	
Yes	2/13	5.94(0.90-39.30)	0.064	4/8	1.05(0.23-4.68)	0.952
**Energy intake (kcal/day)**						
< 1000	22/22	1		17/22	1	
1000–1199	19/19	1.95(0.60-6.35)	0.270	25/33	1.84(0.69-4.87)	0.220
1200–1399	19/25	2.44(0.76-7.81)	0.132	15/39	4.61(1.60-13.29)	0.005
≥ 1400	57/67	1.73(0.60-5.06)	0.313	24/57	5.95(1.90-18.62)	0.002

P for trend[4]	

Abbreviations: aOR, adjusted odds ratio; BMI, body mass index; HDL-C, high density lipoprotein-cholesterol.

[1] Data is expressed as OR (95% confidence interval (CI)), and $p < 0.05$ was considered significantly different.

[2] Multivariable logistic regression were used to examine the associations between the tertile levels of three dietary patterns and breast cancer risk, upon adjusting for age, BMI, education year, family history of breast cancer, smoking, alcohol drinking, physical activity, age at menarche, triglyceride, HDL-C, and energy intake among pre-menopausal women.

[3] Multivariable logistic regression were used to examine the associations between the tertile levels of three dietary patterns and breast cancer risk, upon adjusting for age, BMI, education year, family history of breast cancer, smoking, alcohol drinking, physical activity, age at menarche, triglyceride, HDL-C, and energy intake among post-menopausal women.

[4] *P* for trend was performed through simple linear regression analysis.
